# Factors Attributed to Breastfeeding Success in a Tertiary Obstetric Hospital

**DOI:** 10.1089/whr.2022.0045

**Published:** 2022-07-07

**Authors:** Felicia V. LeMoine, Caitlin Witt, Shelby Howard, Andrew Chapple, LaKedra Pam, Elizabeth F. Sutton

**Affiliations:** ^1^ Department of Obstetrics and Gynecology, Louisiana State University Health Sciences Center, Baton Rouge, Louisiana, USA.; ^2^ Department of Biostatistics, School of Public Health, Louisiana State University Health and Sciences Center, New Orleans, Louisiana, USA.; ^3^ Research Department, Woman's Hospital Research Center, Woman's Hospital, Baton Rouge, Louisiana, USA.

**Keywords:** breastfeeding, obstetrics, prenatal care, pregnancy

## Abstract

**Introduction:**

Increasing breastfeeding rates is a national health objective, however substantial barriers and disparities continue to exist in breastfeeding initiation and continuation. Our study aim is to identify factors associated with birthing persons' breastfeeding “success” (patients admitted to Labor & Delivery desiring to breastfeed and discharged breastfeeding) and breastfeeding “failure” (patients admitted to Labor & Delivery desiring to breastfeed and discharged exclusively formula feeding).

**Materials and Methods:**

We conducted a retrospective cohort study between July 2015 and June 2016. Patients were asked infant feeding plan intentions (breast, formula, combination) upon admission for delivery. Feeding plan was reassessed at discharge from delivery stay and validated to serve as proxy for feeding status at discharge. Logistic regression was used to identify the population(s) most likely to voice intent to breastfeed and to identify predictors of altered breastfeeding intent at discharge.

**Results:**

Between July 2015 and June 2016, 6690 patients met criteria for analysis. Patients reporting intent to breastfeed before delivery were more likely Caucasian (*p* < 0.0001), married (*p* < 0.001), nulliparous (*p* < 0.01), privately insured (*p* < 0.0001), educated (*p* < 0.0001), and older (*p* < 0.01) compared with patients not intending to breastfeed. These characteristics were similar in those who were “successful breastfeeders,” that is, breastfeeding at discharge. The strongest predictor of breastfeeding at discharge was intent to breastfeed before delivery (*p* < 0.0001). African American race was the strongest predictor of nonbreastfeeding intent at admission (*p* < 0.0001) and conversion to formula feeding by hospital discharge (*p* < 0.001).

**Conclusion:**

Intent to breastfeed before delivery was the strongest predictor of breastfeeding at discharge; thus, prenatal breastfeeding education within the at-risk population is crucial to increasing breastfeeding rates.

## Introduction

Infant feeding recommendations by the American Academy of Pediatrics (AAP) advocate exclusive breastfeeding for the first 6 months of life followed by continued breastfeeding through 1 year “or longer as mutually desired by mother and infant.”^1^ This recommendation is substantiated by evidence of reduced risk for communicable and chronic diseases in individuals who were breastfed, including reduction in respiratory tract infection and otitis media rates, gastrointestinal infections, celiac disease, inflammatory bowel disease, asthma, atopic dermatitis, eczema, type 1 and 2 diabetes mellitus, obesity, childhood leukemia and lymphoma, and necrotizing enterocolitis.^2–18^ Maternal benefits from breastfeeding include decreased incidence of postpartum depression, cardiovascular disease in later life, and ovarian and breast cancers.^2,3,19–21^ The American College of Obstetricians and Gynecologists (ACOG) “strongly” supports the AAP recommendation, citing similar evidence for neonatal and maternal benefits.^22,23^

Despite these recommendations and the well-established benefits of breastfeeding, the *2020 Breastfeeding Report Card* published by the Centers for Disease Control reports an overall breastfeeding initiation rate of 84.1% in the United States with only 58.3% and 35.3% of mothers breastfeeding at 6 and 12 months, respectively.^24^ The lowest “ever breastfed” incidence in the United States is in Louisiana at 66.2%. While these rates have slowly risen over the last few decades, there remain notable disparities in breastfeeding rates within geographical regions, among particular races, among different age groups, and among lower educational and socioeconomic levels, which are all barriers to progress in improving breastfeeding incidence.^25–29^

In response to these disparities, Healthy People 2020 included eight breastfeeding initiatives and four subinitiatives.^24^ The Health People 2030 initiatives MICH-15 and MIHC-16 now set aggressive goals for increased proportion of infants breastfed at 6 and 12 months by the year 2030. Accomplishing prolonged breastfeeding goals begins with breastfeeding initiation in the hospital delivery stay. The aim of this study was to identify factors associated with breastfeeding success and failure during hospital stay for delivery; with the ultimate goal to learn with whom and how to intervene to support breastfeeding initiation and continuation.

## Materials and Methods

A retrospective cohort study was performed using electronic medical records of Woman's Hospital, Baton Rouge, Louisiana, from July 1, 2015 to June 30, 2016. This study was approved and monitored by Woman's Hospital Foundation Institutional Review Board. Inclusion criteria included women of Black and White race who delivered at Woman's Hospital during the study period. Patients were excluded due to missing or incomplete data (demographic information or breastfeeding intention), or if not using insurance (i.e., self-pay *n* = 14).

Demographic and obstetrical data collected included age, race, body mass index (BMI), estimated gestational age at delivery, parity (nulliparous vs. multiparous), marital status (married vs. nonmarried, i.e., single, divorced, separated), insurance type (private insurance vs. governmental insurance), education level (less than high school diploma/high school diploma/vocational degree vs. some college/college/graduate degree), time of delivery (night shift 18:00–05:59 vs. day shift 06:00–15:59), route of delivery (vaginal vs. cesarean section), and length of hospital stay (days).

Patients' breastfeeding intention was defined using the question “what is your feeding plan for your baby?” at admission for delivery and at discharge to home. Responses were documented in the electronic medical record by nursing staff. Patient responses were categorized as “intend to breastfeed” if the patient expressed a desire to exclusively breastfeed or a combination of breast and formula feeding. If the patient expressed a desire to only formula feed her baby (i.e., no breastfeeding at all), patients were categorized as “no intention to breastfeed.” Feeding plan was reassessed by nursing staff at the time of hospital discharge from delivery stay as proxy for feeding status at discharge. To confirm that the feeding intention documented at discharge reflected true feeding status at discharge, a validation study was performed using all deliveries from fiscal years 2016–2019 in which breastfeeding intention and actual feeding status at discharge, per nursing documentation of infant feeds, were available from the electronic medical record (*n* = 31,722).

This study demonstrated that 99.7% of infants of mothers who voiced breastfeeding intent at discharge after delivery (i.e., breastfeeding, pumping, or a combination of breast and formula-feeding) were actually receiving breastmilk as indicated by her medical record. Therefore, in this study, it is assumed that an infant received mother's milk (i.e., was breastfed) when the mother reported intent to breastfeed at hospital discharge.

Maternal covariates were compared between maternal intent and no maternal intent to breastfeed using Fisher's exact tests for categorical data and Wilcoxon signed-rank tests for continuous data at hospital admission and at hospital discharge. Patients were then divided into four groups as determined by the patient's pattern of breastfeeding intent throughout hospitalization: intent to breastfeed on admission and at discharge (i.e., “successful breastfeeders”), intent to breastfeed at admission but not breastfeeding at discharge (i.e., “unsuccessful breastfeeders”), no intention to breastfeed at admission and did not breastfeed at discharge (i.e., “never breastfeeders”), and no intention to breastfeed at admission with conversion to breastfeeding at discharge (i.e., “unexpected breastfeeders).

Distributions of maternal covariates were compared between the “successful breastfeeder” and “unsuccessful breastfeeder” groups using a Wilcoxon signed-rank test for continuous data and a chi-squared test of independence for categorical data. The same was done for the “never breastfeeder” and “unexpected breastfeeder” groups. Multivariable logistic regression was performed to predict change of breastfeeding plan and breastfeeding intention at hospital admission and discharge. Covariates were standardized so that adjusted odds ratios (aORs) are directly comparable in magnitude.

## Results

### Cohort characteristics

**FIG. 1.**
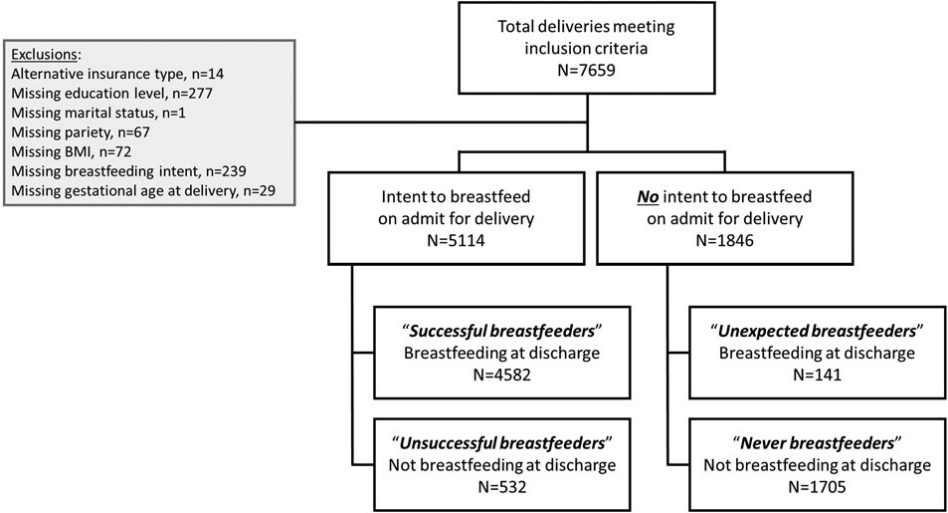
Study flow diagram.

Between July 1, 2015 and June 30, 2016, 7951 deliveries occurred at Woman's Hospital, of which 6960 were included in the analysis (Fig. 1). The population studied was, on average, obese (BMI 33 ± 7 kg/m^2^) and in the mid- to late-20s (age 28 ± 6 years) (Table 1). Half or more of the population was married (50%), privately insured (54%), multiparous (61%), and of Caucasian race (61% vs. 39% African American race). Sixty percent of the population had received at least some college education or greater (Table 1). At the time of admission for delivery, 5114 patients (73%) reported an intent to breastfeed (i.e., expressed a desire to exclusively breastfeed or a combination of breast and formula feed their baby), and 4723 patients (68.9%) were breastfeeding at discharge.

**Table 1.** Characteristics of Overall Patient Population and, Specifically, Characteristics of Those Who Voiced Intent Versus No Intent to Breastfeed at the Time of Hospital Admission

	All *n* = 6960	BF intent *n* = 5114 (73%)	No BF intent *n* = 1846 (27%)	*p*
Maternal age (years)	28 (±6)	28 (±6)	27 (±6)	<0.001
Body mass index (kg/m^2^)	33 (±7)	32 (±7)	33 (±8)	0.149
Married	3462 (50%)	2968 (58%)	494 (27%)	<0.001
Educational level^a^				<0.001
Less than high school	954 (14%)	473 (9%)	481 (26%)	
High school degree	1530 (22%)	940 (18%)	590 (32%)	
Vocational degree	322 (5%)	232 (5%)	90 (5%)	
Some college	1436 (21%)	1084 (21%)	352 (19%)	
College	2094 (30%)	1817 (36%)	277 (15%)	
Graduate degree	624 (9%)	568 (11%)	56 (3%)	
Private insurance	3763 (54%)	3196 (62%)	567 (31%)	<0.001
Nulliparous	2738 (39%)	2234 (44%)	504 (27%)	<0.001
Race				<0.001
African American race	2705 (39%)	1609 (31%)	1096 (59%)	
Caucasian	4255 (61%)	3505 (69%)	750 (41%)	
Route of delivery				0.248
Vaginal delivery	4591 (66%)	3394 (66%)	1197 (65%)	
Cesarean delivery	2369 (34%)	1720 (34%)	649 (35%)	
Night shift delivery	2368 (34%)	1737 (34)	631 (34%)	0.889
GA at delivery (weeks)	38 (±2)	38 (±2)	38 (±2)	<0.001
Maternal length of hospitalization (hours)	71 (±44)	72 (±41)	71 (±52)	0.28

*N* (mean) is reported for categorical variables while mean (±STD) is reported for continuous variables.

^a^ *p-*Value to indicate the comparison between those with less than high school, high school, and vocational degrees versus those with some college, college, or graduate degrees.

BF, breastfeed; GA, gestational age; STD, standard deviation.

### Characteristics and predictors of patients intending to breastfeed before delivery

Patients reporting intent to breastfeed at admission were more likely to be Caucasian, married, nulliparous, privately insured, educated, and slightly older compared with patients not intending to breastfeed (all *p* < 0.01; Table 1). Gestational age at delivery was slightly more advanced in patients who reported intent to breastfeed at admission versus those who voiced no intent to breastfeed (*p* < 0.001). Maternal age and BMI were not different respective to intent versus no intent to breastfeed.

A multivariable logistic regression was performed to predict breastfeeding intent at hospital admission before delivery (results reported as aOR and associated 95% confidence intervals [CI]) (Supplementary Fig. S1). Patients who were married (aOR 1.38, 95% CI 1.28–1.50), nulliparous (aOR 1.48, 95% CI 1.38–1.59), privately insured (aOR 1.18, 95% CI 1.09–1.27), and had some college education or greater (aOR 1.50, 95% CI 1.41–1.60) had a greater likelihood of voicing intent to breastfeed before delivery. Patients of African American race had significantly lower odds of voicing an intent to breastfeed before delivery (aOR 0.71, 95% CI 0.67–0.76).

### Characteristics and predictors of patients with consistent and altered feeding plans

Approximately 10% (*n* = 673) of patients converted their feeding plan between admission for delivery and discharge. Table 2 shows the characteristics of patients who maintained or converted their feeding plan between the time of hospital admission and hospital discharge: “successful breastfeeders,” intended to breastfeed at admission and were breastfeeding at discharge (*n* = 4582, 65.8%); “unsuccessful breastfeeders,” intended to breastfeed at admission but were not breastfeeding at discharge (*n* = 532, 7.6%); “never breastfeeders,” did not intend to breastfeed at admission and were not breastfeeding at discharge (*n* = 1705, 24.5%); and “unexpected breastfeeders,” did not intend to breastfeed at admission but were breastfeeding at discharge (*n* = 141, 2.0%).

**Table 2.** Characteristics of Patients Who Maintained or Converted Breastfeeding Intention Throughout Hospitalization for Delivery

	Successful breastfeeders	Unsuccessful breastfeeders	*p* ^a^	Never breastfeeders	Unexpected breastfeeders	*p* ^b^
*N* (%)	4582 (65.8%)	532 (7.6%)		1705 (24.5%)	141 (2%)	
Maternal age (years)	28.3 ± 5.4	26.0 ± 5.9	<0.001	26.5 ± 5.6	27.6 ± 5.6	0.033
Body mass index (kg/m^2^)	32.3 ± 6.4	33.4 ± 8.0	0.01	32.9 ± 7.7	32.9 ± 6.4	0.58
Married	2807 (61.3%)	161 (30.3%)	<0.001	449 (26.3%)	45 (31.9%)	0.18
Educational level^c^			<0.001			0.034
Less than college	1360 (29.7%)	285 (53.6%)		1082 (63.5%)	79 (56.0%)	
College or higher	3222 (70.3%)	247 (46.4%)		623 (36.5%)	62 (44.0%)	
Private insurance	3005 (65.6%)	191 (35.9%)	<0.001	513 (30.1%)	54 (38.3%)	0.05
Nulliparous	2000 (43.6%)	234 (44%)	0.92	463 (27.2%)	41 (29.1%)	0.69
Race			<0.001			0.05
African American	1307 (28.5%)	302 (56.8%)		1024 (60.1%)	72 (51.1%)	
Caucasian	3275 (71.5%)	230 (43.2%)		681 (39.9%)	69 (48.9%)	
Route of delivery			0.59			0.01
Vaginal delivery	3047 (66.5%)	347 (35.2%)		1120 (35.7%)	77 (54.6%)	
Cesarean delivery	1535 (33.5%)	185 (34.8%)		585 (34.3%)	64 (45.4%)	
Night shift delivery	1553 (33.9%)	184 (34.6%)	0.79	578 (33.9%)	53 (37.6%)	0.427
GA at delivery (weeks)	38.3 ± 1.8	38.2 ± 2.2	0.03	38.2 ± 1.8	36.9 ± 3.3	<0.001
Maternal length of stay (hours)	70.9 ± 39.1	76.5 ± 53.7	<0.001	70.0 ± 45.7	85.7 ± 100.4	0.006

For categorical variables *n* (%) is reported, whereas mean ± STD is reported for continuous variables. Chi-squared tests of independence are used for categorical variables and Wilcoxon signed-rank tests are used for continuous variables.

^a^ *p*-Value pairwise comparisons of successful versus unsuccessful breastfeeders.

^b^ *p*-Value pairwise comparisons of never versus unexpected breastfeeders.

^c^ *p*-Value to indicate the comparison between those with less than high school, high school, and vocational degrees versus those with some college, college, or graduate degrees.

Compared with unsuccessful breastfeeders, successful breastfeeders were more likely to have completed higher levels of education, use private insurance, be married, of Caucasian race, have lower BMI, and be older at admission for delivery (Table 2, all *p* < 0.001). Compared with never breastfeeders, the unexpected breastfeeders were more likely to have completed a higher level of education, be of Caucasian race, and be older at admission for delivery (Table 2, all *p* < 0.05). Interestingly, both groups discharged breastfeeding (successful breastfeeders and unexpected breastfeeders) have significantly longer lengths of stay in the hospital compared with unsuccessful and never breastfeeders, respectively (Table 2, *p* < 0.01).

**FIG. 2.**
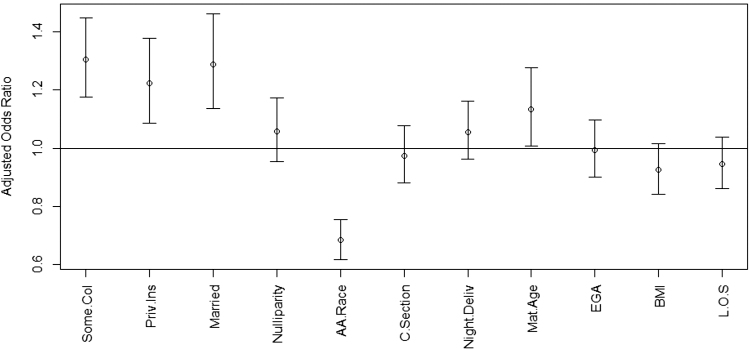
Among patients with breastfeeding intentions at admission for delivery, multivariable logistic regression analysis to predict the influence of covariates on breastfeeding success at discharge. aOR and 95% CI shown. aOR >1 indicated increased likelihood of breastfeeding at discharge, while aOR <1 indicated a decreased likelihood of breastfeeding at discharge in each group. Some.col=some college education or greater; Priv.Ins=privately insured; A.A.Race = African American race; Night.Deliv=delivery between 6 pm and 6 am; Mat.Age=Maternal Age; EGA = estimated gestational age at delivery; L.O.S = maternal length of hospitalization (days) for delivery and postpartum care. aOR, adjusted odds ratio; BMI, body mass index; CI, confidence intervals.

Figure 2 displays the aOR and associated 95% CI for predicting breastfeeding success (i.e., intent to breastfeed at admission with continued breastfeeding intent at discharge). Patients that were married (aOR 1.29, 95% CI 1.14–1.46), had at least some college education or greater (aOR 1.30, 95% CI 1.18–1.45), and used private insurance (aOR 1.22, 95% CI 1.09–1.38) were more likely to report breastfeeding success at discharge. African American mothers were more likely to convert from intent to breastfeed at admission to not breastfeeding at discharge (aOR 0.68, 95% CI 0.62–0.75).

**FIG. 3.**
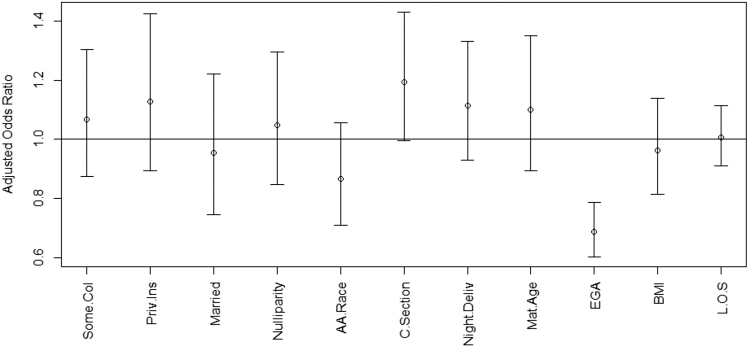
Among patients with no breastfeeding intentions at admission for delivery, multivariable logistic regression analysis to predict the influence of covariates on breastfeeding success at discharge. aOR and 95% CI shown. aOR >1 indicated increased likelihood of breastfeeding at discharge, while aOR <1 indicated a decreased likelihood of breastfeeding at discharge in each group.

Of patients who voiced no intent to breastfeed at admission, Figure 3 displays the aORs for the effect of covariates on their conversion to breastfeeding at discharge. Increased gestational age at delivery (aOR 0.69, 95% CI 0.60–0.79) decreased the likelihood of breastfeeding conversion. Among the patients who voiced no intent to breastfeed before delivery, 25.5% of those with preterm deliveries (<37 0/7 weeks gestation) converted to breastfeeding at hospital discharge, compared with only a 10.4% conversion rate in those with term deliveries. Age, race, BMI, marital status, insurance type, route of delivery, and maternal length of hospital stay had no effect on conversion of breastfeeding intent within our cohort.

### Characteristics and predictors of patients breastfeeding at discharge

**FIG. 4.**
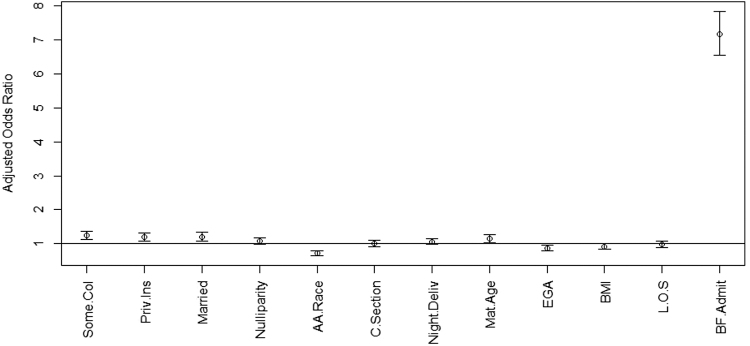
Multivariate logistic regression for predictors of breastfeeding intention at the time of hospital discharge. aOR >1 indicated increased likelihood breastfeeding intention, while aOR <1 indicated a decreased likelihood breastfeeding intention at the time of hospital discharge following delivery. Education levels greater than high school graduate, private insurance, and married patients were more likely to stick with breastfeeding intent. African American mothers were more likely to change their mind from breastfeeding.

Ultimately, a total of 4723 patients (67.9% of the total cohort) were breastfeeding at the time of hospital discharge. Figure 4 displays the aOR and CI associated with predicting breastfeeding status at discharge. Intent to breastfeed at admission was by far the strongest predictor of breastfeeding at discharge (aOR 7.16, 95% CI 6.55–7.84). Also, patients who were educated (aOR 1.25, 95% CI 1.14–1.37), privately insured (aOR 1.19, 95% CI 1.07–1.32), married (aOR 1.20, 95% CI 1.07–1.34), and of increased maternal age (aOR 1.14, 95% CI 1.03–1.27) had a higher probability of breastfeeding at hospital discharge. African American patients (aOR 0.72, 95% CI 0.66–0.79) and patients who delivered at more advanced gestational ages (aOR 0.87, 95% CI 0.80–0.96) were less likely to breastfeed at discharge.

## Discussion

The aim of this study is to improve a practitioner's ability to recognize birthing persons who are “at risk” for unsuccessful breastfeeding and may benefit from proactive intervention. In a retrospective cohort study within an obstetric hospital in Louisiana, our study evaluated predictors of breastfeeding intention at admission for labor and delivery, as well as predictive factors for altered feeding plans before and after birth. We then assessed predictors of breastfeeding at discharge from the delivery hospital stay. A significant finding among this study is that the strongest predictor of a patient breastfeeding at discharge was their intent to breastfeed at the time of admission for delivery. Considering most factors associated with decreased odds for breastfeeding are not modifiable (including those identified herein: race, marital status, education level, and insurance), the potential for changing a patient's intention to breastfeed before admission for delivery holds exciting potential to increase odds of breastfeeding through counseling and education.

Our study is a novel addition to the field for understanding breastfeeding initiation and continuation by considering several subgroupings of the breastfeeding experience grouping cohorts as “successful,” “unsuccessful,” “unexpected,” and “never” breastfeeders according to their feeding intentions before delivery and feeding plan at discharge after delivery. By leveraging feeding intention documentation in the medical record before and after birth, we deconstructed the patient's breastfeeding intention course to better understand who is and is not intending to breastfeed, who is and is not able to sustain those intentions, and how might we recognize “at-risk” persons to deliver proactive intervention and education to support breastfeeding.

Along with intention to breastfeed, our findings show, similar to other reports that socioeconomic status, education level, and poor family or social support are factors that decrease the likelihood of breastfeeding initiation and continuation.^25–29^ Through identification of these at-risk populations, educational and financial support efforts can be targeted specifically within these populations, specifically before delivery. ACOG and AAP recommend that all providers caring for either mother, child, or the mother/child unit (e.g., obstetricians, family medicine providers, pediatricians) be educated and trained on breastfeeding and its associated maternal and neonatal/childhood benefits, and that this information is imparted to patients.^22,23^

Educational efforts should be emphasized within these at-risk populations through earlier introduction of breastfeeding education and frequent re-education throughout the prenatal period. This could be done through the use of current national education efforts (e.g., the “It's Only Natural” campaign from the Office on Women's Health) or through institution-specific campaigns targeted at the at-risk populations.^30^ Similarly, provision of financial support and education on financial benefits of breastfeeding through governmental or institutional programs may also address such population disparities.^31^ It is the ultimate goal that implementation of targeted efforts for cohorts “at risk” for never breastfeeding to increase breastfeeding rates and, subsequently, improve maternal and neonatal outcomes.

Strengths of this study include the geographical setting and diverse population. Performed within a tertiary referral center in south-central Louisiana, Woman's Hospital provided a large and diverse obstetric population for study, which comprised high rates of known risk factors for low breastfeeding rates (e.g., patients of lower socioeconomic status and education levels). Also, while Woman's Hospital earned the “Baby-Friendly” designation in 2018, this had not yet been instituted at the time of the study.^32^ Therefore, rates of breastfeeding intent and breastfeeding success can be argued to primarily be influenced by patient preference. Lack of inpatient support is unlikely as a confounding factor as 24-hour lactation services were available to all patients following delivery (regardless of breastfeeding intent) despite lack of “Baby-Friendly” designation.

Alternatively, the absence of the “Baby-Friendly” designation serves as a limitation as the breastfeeding rate may not accurately reflect post-“Baby-Friendly” breastfeeding rates. With the additional education support and mother-infant support, successful breastfeeding rates may be, theoretically, higher in more recent assessments. Additional limitations of this study include the institution-specific nature of the results obtained and the lack of differentiation between patients exclusively breastfeeding from breast and formula-feeding. To account for these limitations, a large, multi-“ Baby-Friendly” center prospective study assessing breastfeeding intentions at admission and actual infant feeding plan at discharge could support this work.

Future studies targeted at identifying factors influencing exclusive breastfeeding versus breast and formula feeding and factors that influence rates of continued breastfeeding at 6 and 12 months would also be of interest. Assessment of breastfeeding intentions and successful breastfeeding rates among patients with babies admitted to the neonatal intensive care unit would also be of interest. Suggestions for more subjective studies would include assessment of patient explanations for breastfeeding intention conversion with subsequent efforts targeted at these patient-perceived barriers.

## Conclusion

In conclusion, this study identifies that the strongest predictive indicator for breastfeeding success was intention to breastfeed before admission for delivery. This study highlights the populations at increased risk for nonbreastfeeding intention and breastfeeding discontinuation. Ideally, provision of financial and social support along with prenatal and neonatal education centered on the maternal and neonatal benefits, cost-effectiveness, and expectations of breastfeeding, particularly among the nulliparous, government-insured, nonmarried, and African American population, may help mend the disparities seen in breastfeeding rates.

## Supplementary Material

Supplemental data

## Abbreviations Used

AAP: American Academy of Pediatrics
ACOG: American College of Obstetricians and Gynecologists
aOR: adjusted odds ratios
BF: breastfeed
BMI: body mass index
CI: confidence intervals
GA: gestational age
STD: standard deviation
