# Supplementary material for: Factors Attributed to Breastfeeding Success in a Tertiary Obstetric Hospital
Source: Womens Health Rep (New Rochelle). 2022 Jul 7;3(1):624–32. doi: 10.1089/whr.2022.0045 (PMC9518802; doi:10.1089/whr.2022.0045)

**Supplement Figure 1**. Influence of various covariates on intent to breastfeed at the time of admission for delivery. Multivariable logistic regression analysis was performed to determine which covariates influenced the likelihood of a patient voicing intent to breast feed at admission for delivery. Adjusted odds ratio (aOR) greater than 1 indicated increased likelihood of intent to breastfeed at hospital admission while aOR less than 1 indicative of decreased likelihood of intent to breastfeed at hospital discharge following delivery.


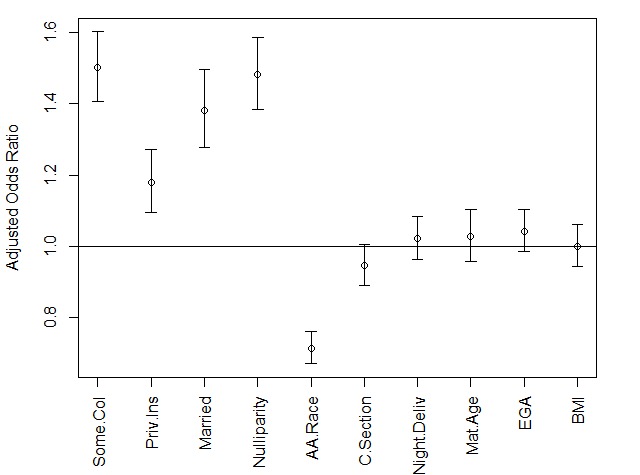

Supplement: Supplemental data [file Supp_FigS1.docx]
